# Upregulated LIMD1 alleviates pressure overload-induced cardiac hypertrophy via inhibits YAP1/AKT/GSK3β signaling

**DOI:** 10.1371/journal.pone.0316149

**Published:** 2025-02-12

**Authors:** Fengwen Xie, Bin Yuan, Ye Zhang, Liru Chen, Yingmei Zhong, Quan Xu

**Affiliations:** 1 Department of Thoracic Surgery, Jiangxi Provincial People’s Hospital, The First Affiliated Hospital of Nanchang Medical College, Nanchang, China; 2 Department of Cardiovascular Surgery, The First Affiliated Hospital of Nanchang University, Nanchang, China; Georgia State University, UNITED STATES OF AMERICA

## Abstract

**Objectives:**

Pathological cardiac hypertrophy plays a significant role in the development and progression of heart failure (HF). LIM Domain Containing 1 (LIMD1) serves as a crucial regulatory factor in protein-protein interactions during cellular signal transduction. This study aims to investigate the specific roles and mechanisms of LIMD1 in pathological cardiac remodeling.

**Methods:**

We employed an adeno-associated virus 9 (AAV9) system to overexpress LIMD1 in the hearts through tail vein injection. C57BL/6 mice underwent transverse aortic constriction (TAC) for four weeks. Cardiac function was assessed using echocardiography, while cardiac remodeling was evaluated through histopathology and molecular techniques.

**Results:**

Our findings demonstrated elevated levels of LIMD1 in murine hearts subjected to TAC treatment and H9c2 cells challenged with angiotensin II (Ang II). Compared with wild-type (WT) mice, those injected with AAV-9-LIMD1 exhibited significantly reduced TAC-induced cardiac dysfunction, hypertrophy, and fibrosis. Mechanistically, both *in vitro* and *in vivo* experiments suggested that the beneficial effects of LIMD1 might be associated with the inhibition of the YAP1/AKT/GSK3β signaling pathway.

**Conclusion:**

In summary, this study is the first to demonstrate the protective effects of LIMD1 against TAC-induced pathological cardiac remodeling. These effects are attributed to the inhibition of the YAP1/AKT/GSK3β signaling pathway.

## 1. Introduction

Pathological cardiac hypertrophy is a compensatory response to prolonged biomechanical stress or volume overload, commonly seen in various cardiovascular diseases including arrhythmias, cardiomyopathy, coronary artery disease, congenital heart disease, hypertension, valvular disease, and cardiotoxicity from anticancer drugs [1]. This form of hypertrophy often precedes overt heart failure (HF) and is considered an independent predictor of mortality from cardiovascular disease. While current treatments such as β-blockers, calcium channel blockers, and angiotensin-converting enzyme inhibitors are widely used once clinical symptoms of HF manifest [2], there remains an urgent need for new therapies that can effectively reverse pathological cardiac hypertrophy.

YAP1 (Yes-associated protein 1) is a crucial transcriptional co-activator in the Hippo signaling pathway, primarily regulated through phosphorylation. When phosphorylated, YAP1 typically remains in the cytoplasm, which prevents its translocation into the nucleus to activate transcription. Consequently, inhibiting YAP1 phosphorylation can increase its nuclear concentration, enhancing its transcriptional activity and affecting the expression of specific target genes [3]. Research indicates that activating YAP1 supports cardiomyocyte growth and survival, potentially mitigating myocardial hypertrophy and HF [4,5]. Elevated YAP1 levels also lead to increased AKT phosphorylation, which inhibits GSK3β, resulting in enhanced FOXM1 expression and contributing to cardiomyocyte hypertrophy and fibrosis [6]. Therefore, targeting YAP1 activation could be a vital approach for reversing pathological myocardial hypertrophy.

LIM domains containing 1 (LIMD1) is a member of the Zyxin protein family, essential for protein-protein interactions during cellular signal transduction. LIMD1 has been identified as a modulator of the Hippo-YAP signaling pathway, with YAP1 serving as a downstream effector of LIMD1 [7,8]. Additionally, LIMD1 is recognized as a prognostic marker for survival in gastric cancer, where it inhibits tumor progression by restraining YAP1 activation [9]. Recent research has also highlighted LIMD1 as a significant biomarker and a potential regulator of inflammation in doxorubicin-induced cardiotoxicity [10]. Despite these advancements, the specific roles and mechanisms of LIMD1 in pathological cardiac remodeling are not fully understood. The primary objective of this study is to investigate whether LIMD1 significantly influences pathological cardiac remodeling through the regulation of YAP activation.

## 2. Methods

### 2.1. Animals

All animal procedures were approved by the Institutional Animal Care and Use Committee of Jiangxi Provincial People’s Hospital (NYLLSC20240621). Adeno-associated virus 9 (AAV9) vectors containing the LIMD1 transcription factor (AAV9-LIMD1) were synthesized by Genechem Co., Ltd (Shanghai, China). The full length of LIMD1 was cloned into the AAV9 shuttle plasmid to generate AAV9-LIMD1, whereas the blank AAV9 vector expressing green fluorescent protein were regarded as the negative control (AAV9-GFP). These vectors were administered through tail vein injections one week before surgery. Subsequently, the mice were then given a single injection of 1 × 10^11^ viral genome particles/mouse (AAV9-GFP or AAV9-LIMD1, diluted in 15 μL PBS) via the tail vein. The efficiency of LIMD1 overexpression was shown in S1 Fig. Following 1-week of AAV9-GFP or AAV9-LIMD1 injection, mice were subjected to transverse aortic constriction (TAC) surgery for consecutive 4-weeks to establish a model of pathological cardiac hypertrophy. The experimental mice were randomly divided into four groups: control (CTL) + AAV9-GFP, CTL + AAV9-LIMD1, TAC + AAV9-GFP, and TAC + AAV9-LIMD1.

### 2.2. Animal model of TAC model

Mice were anesthetized with 2% isoflurane, placed on a Harvard Rodent Ventilator (Harvard Apparatus, Holliston, MA), and positioned on a heating pad to maintain body temperature during surgeries. They received a single subcutaneous injection of bupivacaine (2 mg/kg) prior to surgery. A partial thoracotomy to the second rib was performed, followed by the retraction of the ribs using a chest retractor. After the thymus and fat tissue were separated from the aortic arch, the transverse aorta was identified. Two loose knots were then tied around the transverse aorta between the brachiocephalic and left common carotid arteries. These knots were quickly secured against a 27-gauge needle, which was promptly removed to achieve an approximate aortic diameter of 0.4 mm. In the sham group, the procedure was identical except the aorta was not constricted. Postoperative pain was managed with a single injection of buprenorphine (1 mg/kg) before the animals were returned to their facility.

### 2.3. Echocardiography

Echocardiography analysis was performed under continuous anesthesia using 1.5% to 2% isoflurane and a MyLab30CV (ESAOTE) ultrasound system equipped with a 15 MHz probe. The following parameters were measured from M-mode images obtained from the parasternal short-axis view at the level of the papillary muscles: left ventricular end-systolic diameter (LVESD), left ventricular end-diastolic diameter (LVEDD), left ventricular fractional shortening (LVFS), and left ventricular ejection fraction (LVEF).

### 2.4. Histological analysis

Heart tissues embedded in paraffin were sectioned into 5 μm slices. These slices were then stained with Hematoxylin and Eosin (H&E) to examine heart morphology. For assessing the extent of myocardial fibrosis, Masson’s trichrome staining was performed as per the manufacturers’ instructions. Wheat Germ Agglutinin (WGA, L4895, Sigma Aldrich, USA) staining was utilized to evaluate cardiac hypertrophy. The assessment of cardiac morphology, fibrosis, and hypertrophy was conducted using Image J software (NIH Image, Bethesda, MD, USA).

### 2.5. Cell culture and treatment

H9c2 cells were acquired from the Cell Bank of the Chinese Academy of Sciences (Shanghai, China) and cultured in Dulbecco’s Modified Eagle Medium (DMEM, GIBCO, USA, containing 4500 mg/L D-glucose, L-glutamine, sodium pyruvate, and phenol red) supplemented with 10% fetal bovine serum (FBS, GIBCO, USA). To induce overexpression of LIMD1, cells were transduced with Ad-LIMD1 adenoviral vector, while the Ad-empty vehicle (Ad-EV) served as the control; both vectors were synthesized by GeneChem (Shanghai, China). To elucidate the precise molecular mechanisms involved, the cells were treated with angiotensin II (AngII, 1 μM) for 48 hours, with PY60 (10 μM), a specific activator of YAP, for 24 hours, and with SC79 (4 μg/ml), a specific activator of AKT, also for 24 hours.

### 2.6. Rhodamine phalloidin staining

Rhodamine phalloidin staining was conducted to evaluate the relative cell surface area (CSA) in H9c2 cells. Initially, cardiomyocytes were stained with rhodamine phalloidin (5 μg/ml) for one hour at 37°C. Subsequently, the nuclei were stained with 4′,6-diamidino-2-phenylindole (DAPI) for five minutes in the dark. Fluorescence microscopy was used to observe the staining, employing a BX53 microscope (Olympus, Tokyo, Japan).

### 2.7. Western blotting assay

Total protein was extracted from cardiac tissues of left ventricles and H9c2 cells by cell lysis using RIPA Lysis Buffer (Beyotime Institute of Biotechnology, China). The lysate was centrifuged at 12,000 rpm at 4˚C for 10 min, and the supernatant was transferred to a new tube to quantify total protein using the BCA assay. Subsequently, electrophoresis was conducted with 12% SDS gels followed by transfer onto PVDF membranes (EMD Millipore). Following blocking with 5% (w/v) non-fat dry milk, the membranes were incubated with primary antibodies against LIMD1 (1:500, Abclonal, A17585), phosphorylated YAP1 (1:500, CST, 13008), YAP1 (1:1000, CST, 14074), phosphorylated AKT (1:1000, CST, 9018), AKT (1:2000, CST, 9272), phosphorylated GSK3β (1:1000, CST, 9323), GSK3β (1:2000, CST, 9315), and GAPDH (1:10000, Abcam, Ab181602). After three washes with TBST, the membranes were incubated with an HRP-conjugated secondary antibody at room temperature for one hour. Protein bands were visualized using enhanced chemiluminescence (ECL) detection reagent (Bio-Rad Laboratories). Grayscale values were analyzed using Image Lab software (Bio-Rad, USA).

### 2.8. RT-qPCR

Total RNA was extracted from cardiac tissues of left ventricles and cells using Trizol reagent (Invitrogen). First-strand cDNA synthesis was conducted from the extracted RNA using Prime Script™ RT Master Mix (Takara, Tokyo, Japan). RT-qPCR was then performed in a 25 μL reaction mixture on the CFX96 Real-Time PCR Detection System (Bio-Rad Laboratories), comprising 0.4 μmol/L of primers, 50 ng of cDNA, and 12.5 μL of TB Green Premix Ex Taq II (Takara). The expression levels of the target genes were normalized against the levels of beta-actin, which served as an endogenous internal control. Primer sequences are detailed in Table 1.

**Table 1:** Mouse primers for RT-PCR.

Gene	Forward primers	Reverse primers
ANP	ACCAAGGGCTTCTTCCTCT	TTCTACCGGCATCTTCTCC
BNP	AGAACAATCCACGATGCAGAAG	AAACAACCTCAGCCCGTCACA
CTGF	GGGCTTTCGCTTCAGTGCT	GCACTTTTTGCCCTTCTTAATGTT
TGF-β	TGGTCCTCTGGGCATTGC	TCGGTTCATGTCATGGATGGT
GAPDH	CCTCTATGCCAACACAGTGC	GTACTCCTGCTTGCTGATCC

### 2.9. Statistical analysis

For the purpose of statistical analysis, all quantitative data are represented as mean values ± SEM, derived from a minimum of three independent experiments. The normality of data distribution was initially assessed using the Shapiro-Wilk normality test. To ascertain statistical disparities, a two-tailed unpaired Student’s t-test was employed for comparisons between two distinct groups. In cases involving comparisons among three or more groups, statistical significance was evaluated using one-way ANOVA or two-way ANOVA (applicable for analyses with two factors), with subsequent adjustments for multiple comparisons made through Bonferroni’s post hoc test. A threshold of *P < 0.05 was established as the criterion for statistical significance. The statistical analyses were conducted utilizing GraphPad Prism version 8.0 and SPSS software, version 19.0.

## 3. Results

### 3.1. LIMD1 was upregulated under cardiac hypertrophy condition

**Fig 1:**
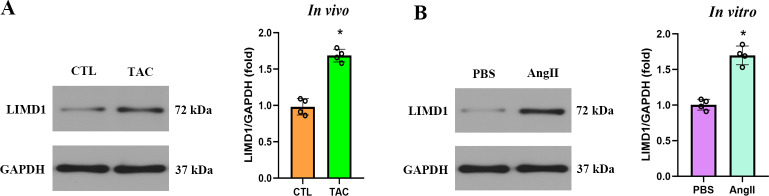
LIMD1 was up-regulated in cardiac hypertrophy condition. **(A)** Representative western blot and quantitative data of LIMD1 protein expression in mice (n = 4 per group). **(B)** Representative western blot and quantitative data of LIMD1 protein expression *in vitro* (n = 4 per group). * *P* < 0.05.

To investigate the role of LIMD1 in cardiac hypertrophy, we analyzed its protein expression under conditions of hypertrophy. Western blot analysis demonstrated that in the ***in vivo*** model, LIMD1 expression was quantitatively increased by approximately 1.75-fold in TAC-treated animals compared to sham-operated controls (Fig 1A). For the ***in vitro*** experiments, LIMD1 protein levels showed a similar trend, with a 1.70-fold increase in cells exposed to AngII that mimic the TAC environment, compared to controls (Fig 1B).

### 3.2. LIMDl overexpression improved TAC-induced cardiac dysfunction

**Fig 2:**
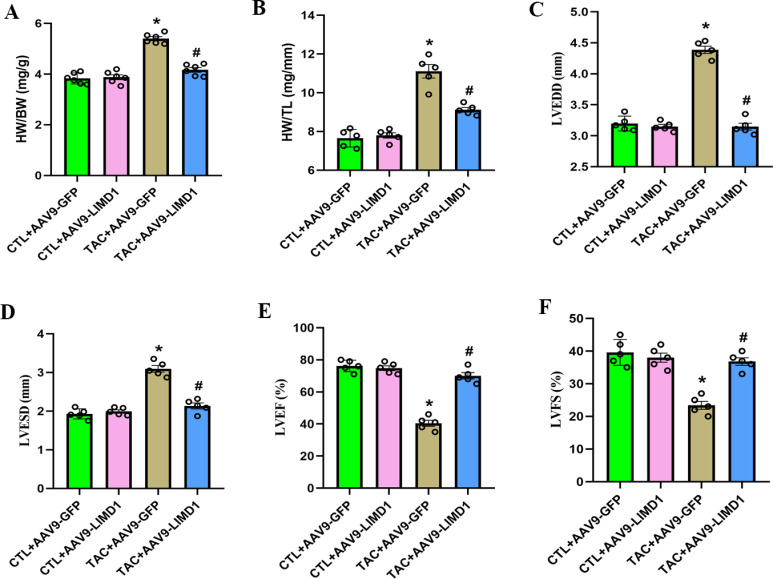
LIMD1 overexpression alleviated TAC-induced cardiac dysfunction *in vivo.* **(A)** Statistical results of heart weight to body weight (HW/BW) ratio (n = 5 per group). **(B)** Statistical results of heart weight to tibia length (HW/TL) ratio (n = 5 per group). **(C and D)** Statistical results of LVEDD and LVESD (n = 5 per group). **(E and F)** Statistical results of LVEF and LVFS (n = 5 per group). * *P* < 0.05 vs. CTL + AAV9-GFP group. #*P* < 0.05 vs. TAC + AAV9-GFP group.

We also examined the impact of LIMD1 overexpression on TAC-induced cardiac dysfunction. Four weeks post-TAC surgery, mice exhibited increased heart weight (HW) to body weight (BW) and HW to tibia length (TL) ratios compared to CTL mice. Overexpression of LIMD1 significantly reduced these ratios, as evidenced in Fig 2A and 2B. Echocardiographic assessments showed significant increases in LVEDD and LVESD in the TAC + AAV9-GFP group versus the CTL + AAV9-GFP group. However, LIMD1 overexpression notably reversed these changes, as illustrated in Fig 2C and 2D. Additionally, significant reductions in LVEF and LVFS were observed in the TAC + AAV9-GFP group, with a reversal of these effects following LIMD1 overexpression, shown in Fig 2E and 2F .

### 3.3. LIMD1 overexpression attenuated TAC-induced cardiac hypertrophy and fibrosis

**Fig 3:**
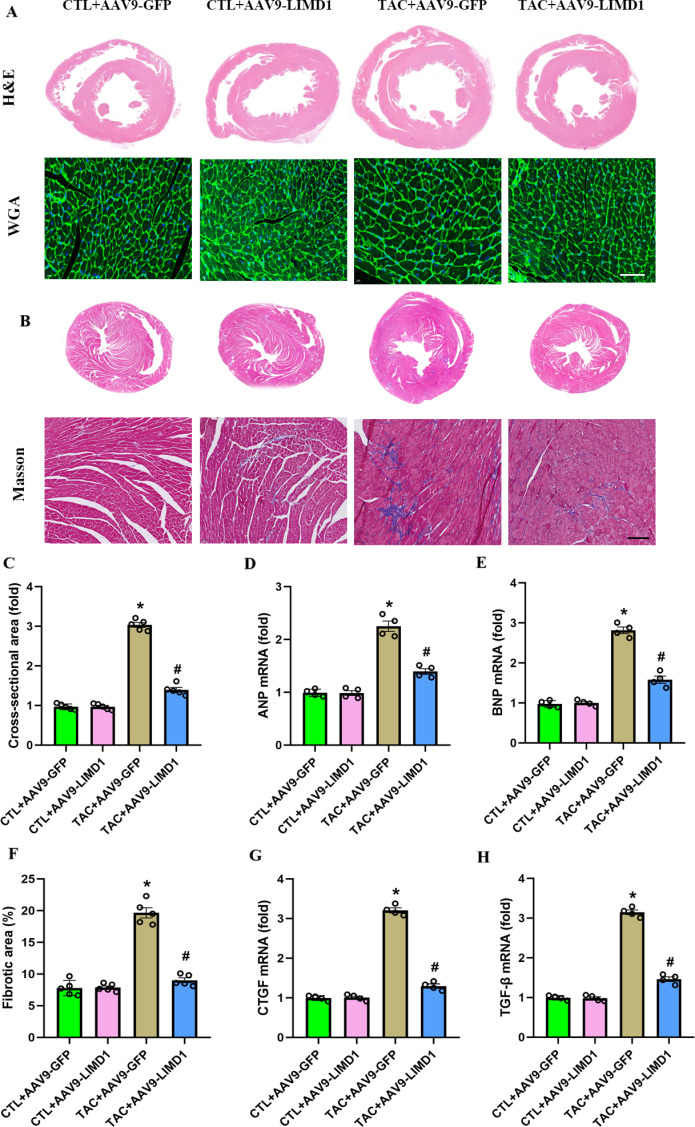
LIMD1 overexpression attenuated DOX-induced cardiac hypertrophy and fibrosis *in vivo.* **(A and B)** Representative images WGA-stained heart sections and quantitative results of cross-sectional area (n = 5 per group). **(C and D)** Quantitative results of mRNA expression of ANP and BNP in heart tissues (n = 4 per group). **(E and F)** Representative images Masson-stained heart sections and quantitative results of fibrotic area (n = 5 per group). **(G and H)** Quantitative results of mRNA expression of TGF-β and CTGF in heart tissues (n = 4 per group). * *P* < 0.05 vs. CTL + AAV9-GFP group. #*P* < 0.05 vs. TAC + AAV9-GFP group. Bar = 50 μm.

We further investigated the effects of LIMD1 overexpression on TAC-induced cardiac hypertrophy and fibrosis. WGA staining revealed that, compared to the CTL + AAV9-GFP group, the TAC + AAV9-GFP group exhibited significantly increased cross-sectional areas, as shown in Fig 3A and 3B. This hypertrophic response was corroborated by RT-PCR analyses, which showed significantly elevated levels of ANP and BNP mRNA in TAC + AAV9-GFP hearts (Fig 3C and 3D). Additionally, Masson’s trichrome staining indicated a significant increase in cardiac fibrosis in the TAC + AAV9-GFP hearts compared to the CTL + AAV9-GFP group (Fig 3E and 3F). This finding was further supported by RT-PCR results, which demonstrated significantly higher CTGF and TGF-β mRNA levels in the TAC + AAV9-GFP group (Fig 3G and 3H). Remarkably, all these changes indicative of cardiac hypertrophy and fibrosis induced by TAC were significantly reversed by overexpressing LIMD1. These findings suggest that LIMD1 overexpression provides protective effects against TAC-induced cardiac hypertrophy and fibrosis.

### 3.4. YAP1/AKT/GSK3β signaling contributes to the cardioprotective effect LIMD1 overexpression

**Fig 4:**
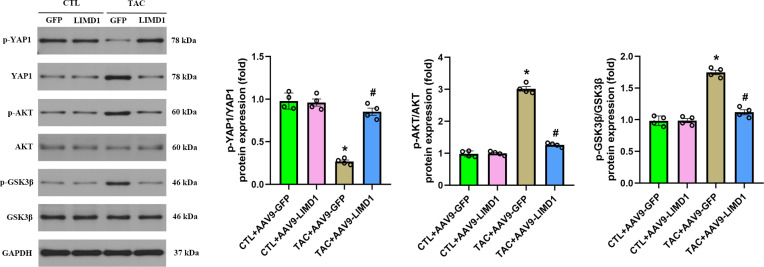
YAP1/AKT/GSK3β signaling contributes to the cardioprotective effect LIMD1 overexpression *in vivo*. Representative western blot and quantitative data of p-YAP1, YAP1, p-AKT, AKT, p-GSK3β and GSK3β protein expression (n = 4 per group). * *P* < 0.05 vs. CTL + AAV9-GFP group. #*P* < 0.05 vs. TAC + AAV9-GFP group.

The YAP1/AKT/GSK3β signaling pathway plays a significant role in cardiac hypertrophy. However, the impact of LIMD1 overexpression on this pathway, particularly when induced by TAC, remains unexplored. We investigated the YAP1/AKT/GSK3β pathway using western blot analysis (Fig 4). Compared to CTL + AAV9-GFP hearts, TAC + AAV9-GFP hearts exhibited significantly decreased p-YAP1 and increased total YAP1, p-AKT, and p-GSK3β. All these alterations induced by TAC were significantly reversed following LIMD1 overexpression.

### 3.5. Activating the YAP1/AKT/GSK3β signaling offset the cardioprotective effect LIMDl overexpression on AnglI-induced cardiac hypertrophy

**Fig 5:**
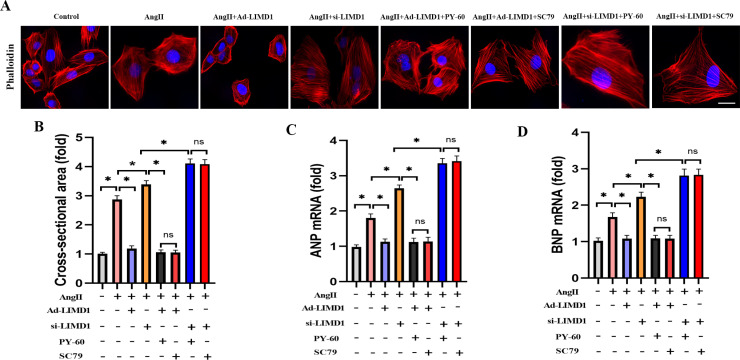
Activating the YAP1/AKT/GSK3β signaling offset the cardioprotective effect LIMDl overexpression on AnglI-induced cardiac hypertrophy *in vitro.* **(A and B)** Representative images of Phalloidin-stained H9c2 cells and quantitative results of cross-sectional area (n = 5 per group). **(C and D)** Quantitative results of mRNA expression of ANP and BNP in H9C2 cells (n = 5 per group). * *P* < 0.05. Bar = 50 μm.

To further validate the role of the YAP1/AKT/GSK3β signaling in cardiac hypertrophy, we employed H9c2 cells to elucidate the underlying mechanisms. Fig 5A and 5B show that the CSA increased in the control group, but significantly decreased in H9c2 cells treated with Ad-LIMD1. Additionally, treatment with PY-60 (1 μM), a specific YAP activator, led to a significant increase in CSA, countering the protective effects of LIMD1 overexpression. Similarly, SC79 (1 μM), a specific AKT activator, also increased CSA, further offsetting LIMD1’s protective role (Fig 5A and 5B). Quantitative RT-PCR analysis of ANP and BNP mRNA expression corroborated these findings. Both YAP1 and AKT activation negated the protective effects of LIMD1 overexpression on AngII-induced cardiac hypertrophy (Fig 5C and 5D). These results underscore the crucial role of YAP1/AKT/GSK3β signaling in AngII-induced cardiac hypertrophy and reveal how activation of this pathway can mitigate the protective effects of LIMD1 overexpression.

## 4. Discussion

Pathological cardiac hypertrophy, induced by pressure overload, is closely linked with chronic HF. Consequently, inhibiting pathological cardiac hypertrophy is considered a critical strategy to prevent the irreversible progression to end-stage HF. In this study, we observed a significant upregulation of LIMD1 expression in mice with HF induced by pressure overload and in H9c2 cells stimulated by AngII. Overexpression of LIMD1 was able to reverse excessive cardiac hypertrophy and fibrosis in HF mice, as well as improve cardiac function. Mechanistically, both *in vivo* and *in vitro* experiments demonstrated that overexpression of LIMD1 significantly inhibits the activation of the YAP1/AKT/GSK3β signaling pathway, thereby reversing pathological cardiac hypertrophy induced by pressure overload. Thus, our findings suggest that LIMD1 is a crucial target for the prevention and treatment of HF progression.

Previous studies have predominantly explored the role of LIMD1 in immunity and tumor progression [10–12]. Additionally, LIMD1 is implicated in modulating the cardiotoxic effects induced by doxorubicin [10]. However, the role of LIMD1 in pathological cardiac hypertrophy remains poorly understood. In this study, we observed a significant upregulation of LIMD1 expression in hypertrophic cardiomyocytes, and found that overexpression of LIMD1 mitigated the progression of cardiac hypertrophy. These findings suggest that the hypertrophy-induced increase in LIMD1 protein acts as a compensatory, albeit insufficient, response to cardiac injury. Moreover, gain-of-function studies have clarified the protective role of LIMD1 in inhibiting the progression of pathological cardiac hypertrophy.

Cardiac fibrosis is a critical pathological change resulting from pressure overload-induced left ventricular dysfunction, contributing significantly to the progression of HF [13,14]. The spatial and functional proximity of cardiac fibroblasts to cardiomyocytes is crucial for maintaining cardiac function in a healthy heart and exacerbating cardiac dysfunction in disease states. In this study, we observed that the upregulation of LIMD1 significantly attenuated myocardial fibrosis in HF mice. This finding is consistent with previous research showing that LIMD1 modulates the activation of cardiac fibroblasts [15]. Therefore, overexpression of LIMD1 mitigates pathological myocardial remodeling induced by TAC, thereby influencing the onset and progression of HF.

Research has established that the activation of cellular molecular signaling pathways can promote maladaptive cardiac remodeling and dysfunction, ultimately leading to HF. Consequently, targeted modulation of these pathways represents a critical therapeutic strategy for managing HF [2]. Prior studies have underscored the central role of the Hippo-YAP pathway in regulating cardiomyocyte proliferation and differentiation, stress response, and mechanical signaling. Thus, targeting the Hippo-YAP pathway holds significant promise for developing innovative therapeutic approaches for cardiac repair and regeneration in cases of refractory HF [16]. LIMD1 has been shown to negatively modulate Hippo-YAP signaling activity [7,8]. A recent study revealed that upregulation of YAP1 enhances AKT phosphorylation, which in turn inhibits GSK3β, leading to increased FOXM1 expression. This sequence contributes to cardiomyocyte hypertrophy and fibrosis [6]. Extensive research has demonstrated that inhibiting the AKT/GSK3β signaling pathway can reduce pathological cardiac hypertrophy caused by pressure overload [17,18]. In this context, our study explored the effects of LIMD1 on the YAP1/AKT/GSK3β signaling pathway. We discovered that overexpression of LIMD1 attenuates this signaling, thus mitigating pathological cardiac hypertrophy. Aligning with these observations, the inhibition of YAP1 activation has been shown to alleviate pathological hypertrophy in mice subjected to chronic pressure overload via abdominal aortic constriction [4]. Therefore, our findings suggest that LIMD1 improves outcomes in post-HF pathological cardiac hypertrophy by targeting the YAP1/AKT/GSK3β signaling pathway.

## 5. Limitations

The current study has several limitations. Firstly, employing cardiac-specific LIMD1 overexpression or knockout models could provide deeper insights into its protective role against pathological cardiac hypertrophy. Secondly, using human-induced pluripotent stem cells could enhance our understanding of LIMD1’s function in hypertrophy and facilitate its clinical translation. Thirdly, translating findings from genetically modified mice to humans presents significant challenges. Therefore, to substantiate our conclusions on the translational potential of HF regulators, future research should include experiments using human samples.

## 6. Conclusions

Our findings demonstrate that overexpressing LIMD1 mitigates cardiomyocyte hypertrophy by suppressing YAP1 and the subsequent AKT/GSK3β signaling pathways. Diminishing YAP1 activation can effectively reduce the hypertrophy exacerbated by LIMD1 overexpression. This research elucidates the regulatory dynamics and mechanisms of LIMD1 under pathological conditions, suggesting its potential as a therapeutic target for pathological cardiac hypertrophy.

## Supporting information

S1 Fig The efficiency of LIMD1 overexpression by AAV9-LIMD1. (TIF)

S1 Data Raw western-blot image. (PDF)

S2 Data Raw data used in the manuscript. (XLSX)
