# Supplementary figures and images for: Upregulated LIMD1 alleviates pressure overload-induced cardiac hypertrophy via inhibits YAP1/AKT/GSK3β signaling
Source: PLoS One. 2025 Feb 12;20(2):e0316149. doi: 10.1371/journal.pone.0316149 (PMC11819601; doi:10.1371/journal.pone.0316149)

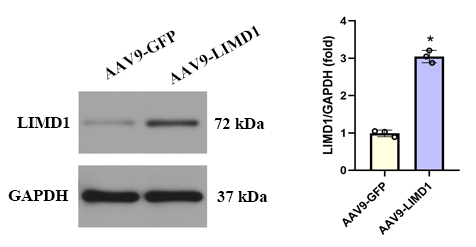

Supplement: S1 Fig — (TIF) [file pone.0316149.s001.tif]

**Fig.1A**

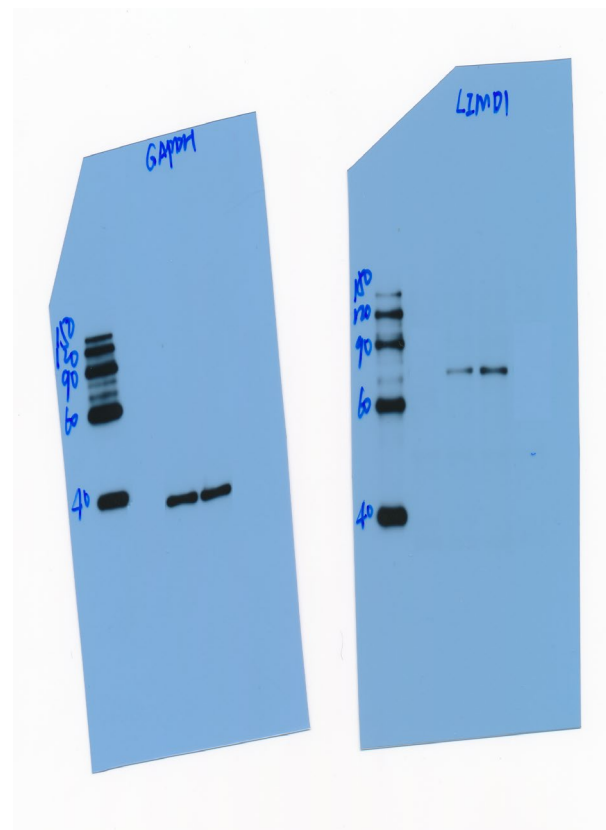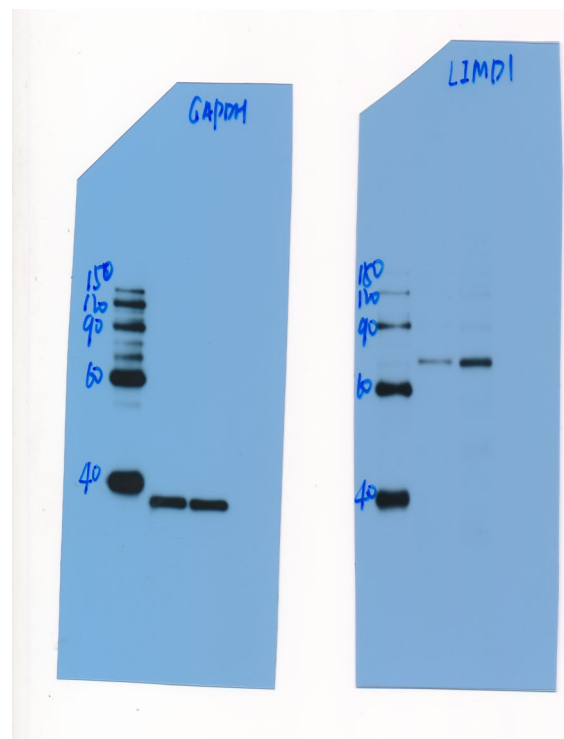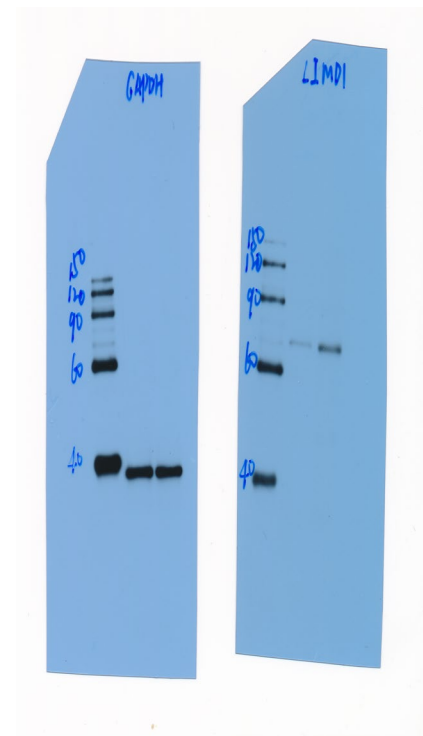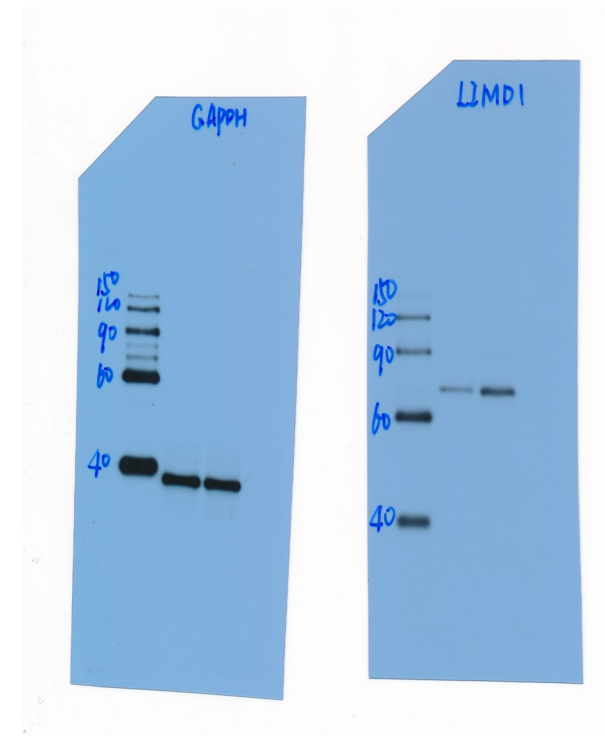

**Fig.1B**

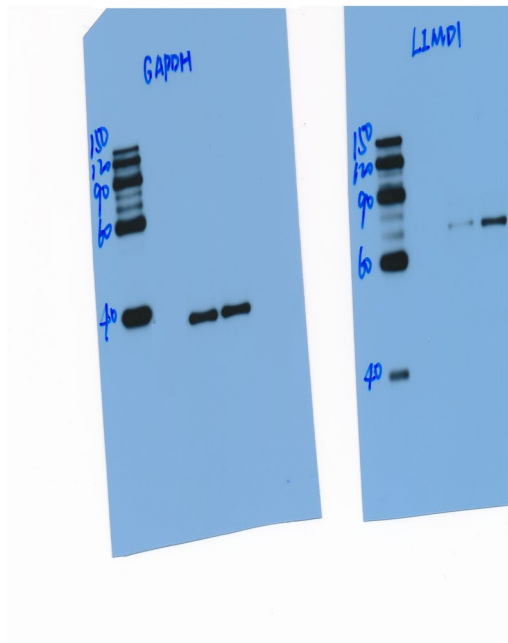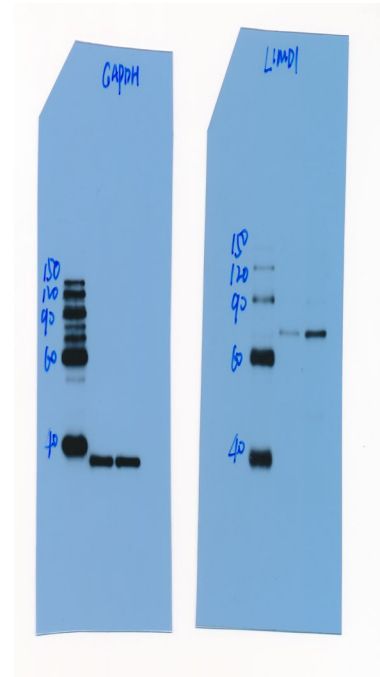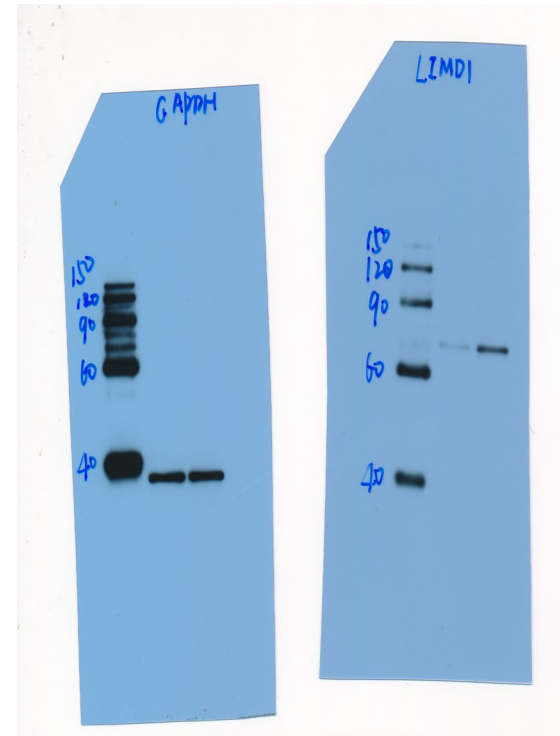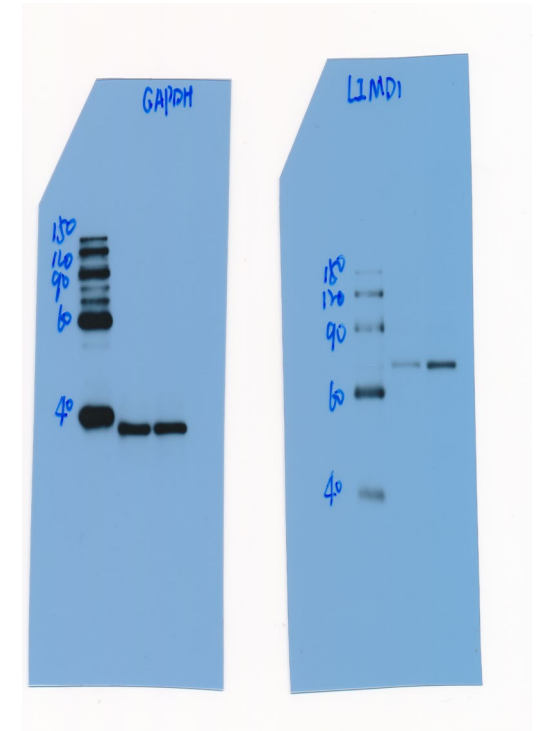

Fig.5

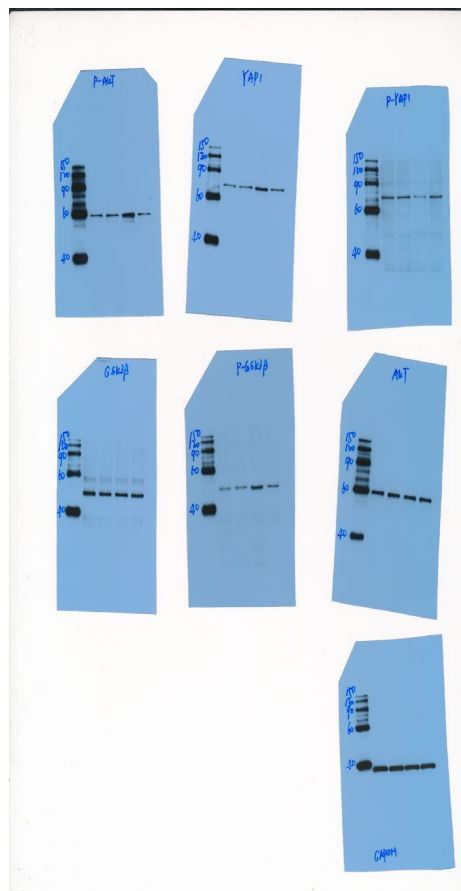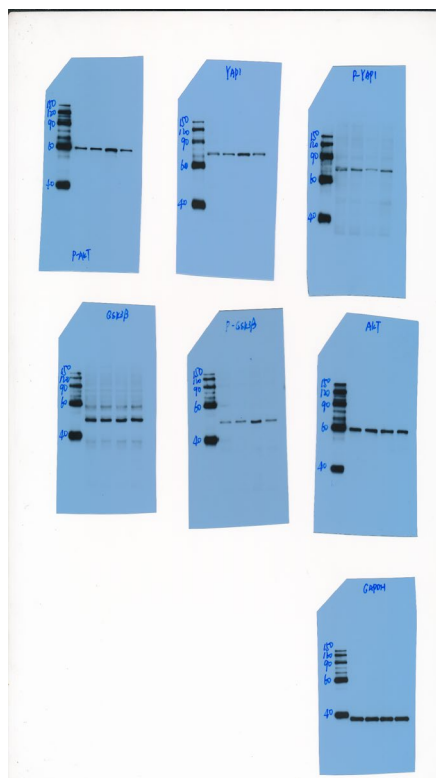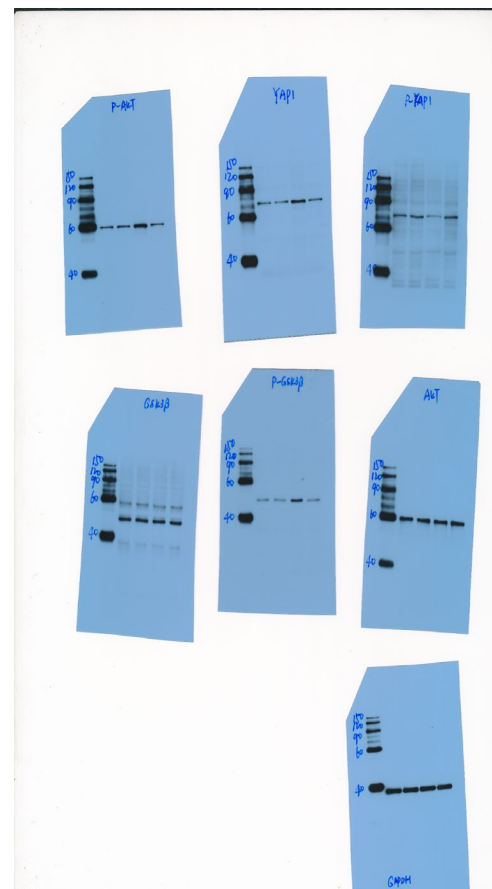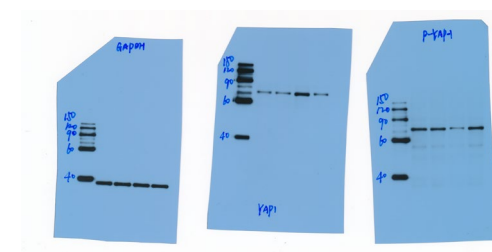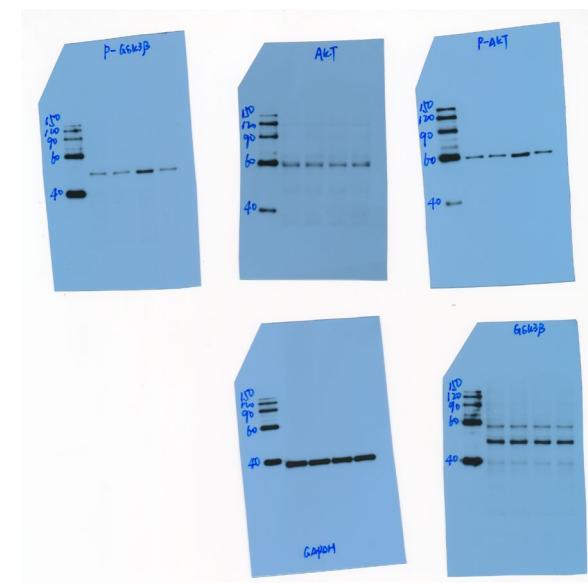

Supplement: S1 Data — (PDF) [file pone.0316149.s002.pdf]
